# Investigating the effect of N-doping on carbon quantum dots structure, optical properties and metal ion screening

**DOI:** 10.1038/s41598-022-16893-x

**Published:** 2022-08-15

**Authors:** Kiem Giap Nguyen, Ioan-Alexandru Baragau, Radka Gromicova, Adela Nicolaev, Stuart A. J. Thomson, Alistair Rennie, Nicholas P. Power, Muhammad Tariq Sajjad, Suela Kellici

**Affiliations:** 1 London Centre for Energy Engineering (LCEE), School of Engineering, London South Bank University, 103 Borough Road, London, SE1 0AA UK; 2 School of Life Health and Chemical Sciences, Open University, Walton Hall, Milton Keynes, UK; 3 National Institute of Materials Physics, Atomistilor 405A, 077125 Magurele, Ilfov Romania; 4 Edinburgh Instruments Ltd., 2 Bain Square, Livingston, EH54 7DQ UK

**Keywords:** Quantum dots, Synthesis and processing

## Abstract

Carbon quantum dots (CQDs) derived from biomass, a suggested green approach for nanomaterial synthesis, often possess poor optical properties and have low photoluminescence quantum yield (PLQY). This study employed an environmentally friendly, cost-effective, continuous hydrothermal flow synthesis (CHFS) process to synthesise efficient nitrogen-doped carbon quantum dots (N-CQDs) from biomass precursors (glucose in the presence of ammonia). The concentrations of ammonia, as nitrogen dopant precursor, were varied to optimise the optical properties of CQDs. Optimised N-CQDs showed significant enhancement in fluorescence emission properties with a PLQY of 9.6% compared to pure glucose derived-CQDs (g-CQDs) without nitrogen doping which have PLQY of less than 1%. With stability over a pH range of pH 2 to pH 11, the N-CQDs showed excellent sensitivity as a nano-sensor for the highly toxic highly-pollutant chromium (VI), where efficient photoluminescence (PL) quenching was observed. The optimised nitrogen-doping process demonstrated effective and efficient tuning of the overall electronic structure of the N-CQDs resulting in enhanced optical properties and performance as a nano-sensor.

## Introduction

The demand for high-performance carbon quantum dots (CQDs) with a range of applications, including sensing has been steadily increasing. However, the synthesis of CQDs continues to face challenges including high costs, lengthy multistep processes, and the use of hazardous substances^1,2^. Recently, biomass-derived CQDs have attracted considerable attention, and are considered as an optimal and green approach to prepare efficient CQDs. Biomass and biomass waste (agriculture product, agricultural residue, municipal solid waste etc.) are abundant, high in carbon content (45–55%), and are an environmentally friendly renewable resource^3^. Therefore, the utilisation of biomass as carbon resources for nanomaterial synthesis is an eco-friendly process and expected to reduce the total synthetic cost^4^. Although a broad range of biomass materials have been employed in producing CQDs, generally, these synthetic routes faced problems associated with poor control of the CQDs particle size, quality, and homogeneity of the product^5^. In addition, the CQDs synthesised from biomass or biomass waste, commonly possess poor optical properties and a low PLQY. The doping of heteroatom such as (N, P, S) is one of the most common methods to improve the optical properties of biomass-derived CQDs^6,7^. However, the questions related to the origin of the optical improvement with optimised dosing of these heteroatoms still need to be answered. Furthermore, in most conventional methodologies, these doping processes result in a longer synthesis time and higher energy consumption^8^.

In this work, the continuous hydrothermal flow synthesis (CHFS) which is primarily water-based was employed; thus, it is considered the greenest and most promising synthesis method for making CQDs. Notably, the CHFS allows designing or tailoring of the nanoparticles for specific functions based on the nucleation and surface functional processes. The comparison between CHFS and the traditional hydrothermal process revealed that the CHFS consumed less energy and time, while producing a highly homogenous quality product^9^. Moreover, the continuous hydrothermal process can be employed in multi-purposes such as controlling the nucleation to control the particle size and the addition of surfactant coating or dopant without further post-treatments^10^. In this paper, we report the use of CHFS process, to successfully synthesise N-doped carbon quantum dots (N-CQDs) from glucose which is an abundant, readily available, cost-effective biomass carbon source; and ammonia is used as a nitrogen dopant. Synthesised N-CQDs with different concentrations of ammonia were used to explore the effect of the concentration of nitrogen dopant on the optical characteristics of CQDs. A range of characterisation techniques were employed to investigate the origin of the optical enhancement. The performance of the N-CQDs as chemical nanosensor was tested. Currently clean water resources are foremost among global challenges facing society today. A significant proportion of the worlds wastewater containing heavy metals as pollutants  is disposed untreated in to the environment^11^. Therefore, the application of the prepared N-CQDs as chemical sensor to detect chromium (VI) which is carcinogenic, hemotoxic, and genotoxic; the main source being industrial waste water, would be timely.

## Experimental work

### Chemicals

Glucose, ammonia (32%), potassium chromate, and potassium dichromate were purchased from Fisher Scientific. The solutions of metal ions used for the sensing application experiments were prepared using nitrate (Ag^+^, Ce^3+^, Co^2+^, Cr^3+^, Ni^2+^, Fe^3+^), sulphate (Cu^2+^ and Fe^2+^), chloride (K^+^, Na^+^, Mg^2+^) and sodium (CrO_4_^2−^, Cr_2_O_7_^2−^_,_ NO_3_^−^, CH_3_COO^−^, HCOO^−^, SO_4_^2−^, F^−^, Cl^−^, Br^−^, I^−^). These chemicals were purchased from Sigma-Aldrich and were used as received. 15 MΩ deionized H_2_O (ELGA Purelab) was used for all the experiments.

### Equipment

UV–Vis spectrophotometry: Shimadzu UV-1800 was used to perform the absorption measurements (λ = 200 to 800 nm) using a quartz cuvette (10 mm).

Photoluminescence (PL) spectroscopy: The steady-state fluorescence spectra of NCQDs were measured with Shimadzu RF-6000 spectrofluorophotometer.

High-resolution transmission electron microscopy (HRTEM): NCQDs were diluted in isopropanol and applied onto a carbon holey mesh grid (Agar) and allowed to air dry. The samples were then imaged using JEM 2100 (Jeol, Japan) at an acceleration voltage of 200 kV and at a range of magnifications between 15 and 500 K. Representatives NCQDs samples (g-CQDs, N-0.25, N-1, N-5 and N-10) were imaged and analysed.

Fourier–transform infrared (FTIR) spectra was recorded using an IR Affinity-1S Fourier transform infrared spectrometer instrument.

Raman spectra of the prepared N-CQDs was measured with a Horiba LabRAM HR Evolution spectrometer with radiation at 633 nm.

An Edinburgh Instruments FLS1000 photoluminescence spectrometer was used to measure the PL lifetime and the PLQY of the samples. The lifetime was measured using a 375 nm pulsed laser, and the data was fitted with 3-exponentials after reconvolution with instrument response function. The absolute quantum yield (QY^abs^) of the samples were investigated by using integrating sphere accessory with a standard method. Then, the true fluorescence quantum yield (QY^true^) is calculated by using the Eq. (1) where a is the fraction of the re-absorbed area.

1 $${\mathrm{QY}}^{\mathrm{true}}=\frac{{\mathrm{QY}}^{\mathrm{abs}}}{1-\mathrm{a}+\mathrm{a}.\frac{{\mathrm{QY}}^{\mathrm{abs}}}{100}}$$

X-ray photoelectron spectroscopy (XPS): an AXIS Ultra DLD (Kratos Surface Analysis) setup equipped with a 180° hemispherical analyser, using Al K_α1_ (1486.74 eV) radiation produced by a mono-chromicized X-ray source at operating power of 300 W (15 kV × 20 mA), with spot size of 0.7 mm was used to record the XPS spectra. The partial charge compensation was achieved by using a flood gun operating at 1.52 A filament current, 2.73 V charge balance, and 1.02 V filament bias. The vacuum in the analysis chamber was at least 1 × 10^−8^ mbar.

### Synthetic methodology

Continuous hydrothermal flow synthesis (CHFS) was employed to synthesize N-CQDs (Supplementary Fig. 1). The process consists of three feedstock streams; (i) glucose (with a concentration of 70 mg mL^−1^) which was used as carbon source, (ii) ammonia with varied concentrations from 0.25 M up to 10.0 M was used as an N-dopant, and (iii) supercritical water which is the key parameter of this reaction. Firstly, the deionized water (with the flow rate 20 mL min^−1^) was heated up to 450 °C, and the pressure was kept at 24.8 MPa by using a back-pressure regulator (BPR) during the experiment (this condition is previously reported by our group as the optimised environment for the CQDs synthesis)^10^. The reaction was conducted by injecting the two precursors into the engineered mixer labelled as the “Reactor” (Fig. S1). Here, the precursors were mixed with supercritical water, and the nano dots were produced (in fraction of seconds). The residence time (~ 1.8 s) of the reaction was controlled by the flow rate of the precursors; both glucose and ammonia were pumped at the same time into the reactor with 5 mL min^−1^ flow rate. The reaction mixture travelled through a cooler to the BPR, and was collected for further treatments. The obtained solutions from the CHFS reaction mixtures were filtered using a 0.2 µm alumina membrane; subsequently, the solutions were continuously dialysed using a 30 kDa membrane in a tangential filtration unit. The cleaned solutions were freeze-dried, and the obtained average yield was 10.68 mg ml^−1^.

The CQDs samples synthesised from [glucose] = 70 mg mL^−1^ and ammonia with varied concentrations (0.25 M, 0.5 M, 0. 75 M, 1.0 M, 2.5 M, 5.0 M, 7.5 M and 10.0 M), were denoted as N-0.25, N-0.5, N-0.75, N-1, N-2.5, N-5, N-7.5, and N-10, respectively; g-CQDs was synthesised from the same source (glucose) but without nitrogen doping. The fluorescent photographs of the samples are shown in Fig. S2.

### pH stability testing

The solutions with pH ranging from 1 to 13 were prepared using varying concentrations of NaOH (initial concentration 1.0 M) and HCl (1.0 M) solutions which were then diluted to the required pH. A dilute solution of N-CQDs (optical density, OD = 0.1) in deionised water was prepared. Following that, 100 μL of this diluted N-CQDs solution was added to a 3000 μL of each solution prepared at thedesired pH level (range 1–13). A pH meter was used to measure the corresponding pH values. The fluorescence spectra of these solutions were recorded using a Shimadzu RF-6000 Spectro fluorophotometer.

### Chromium (VI) ion-sensing experiments

The detection of Cr (VI) ion experiment was conducted with various metal ions (as reported above), each prepared with a concentration of 50 ppm. In a typical experiment, 100 μL, N-CQDs (0.1 OD) was added to the 3.0 mL aqueous metal ion solution. The fluorescence spectrum of mixture solutions was measured using a Shimadzu RF-6000 Spectro fluorophotometermeas. The fluorescence lifetime was investigated to achieve a deeper understanding of the quenching mechanism using an Edinburgh Instruments FLS1000 spectrophotometer.

### Limits of detection (LOD) and limits of quantification (LOQ)

The sensitivity of the N-CQDs sensor for Cr (VI) was investigated by evaluating their LOD and LOQ. For that, Cr (VI) ion solutions with various concentrations of 300 ppm, 200 ppm, 100 ppm, 50 ppm, 30 ppm, 10 ppm, 5 ppm, 2 ppm, 1 ppm and 0.5 ppm were first prepared. Then, 100 μL of N-CQDs were added to 3.0 mL of the prepared Cr (VI) ion solutions. The fluorescence spectra were recorded to estimate the LOD and LOQ by using Stem-Volmer graphs, LOD = 3σ/K_sv_, LOQ = 10σ/K_sv_, where K_sv_ is the slope of the graph, and σ is the error of the intercept.

## Results and discussion

**Figure 1:**
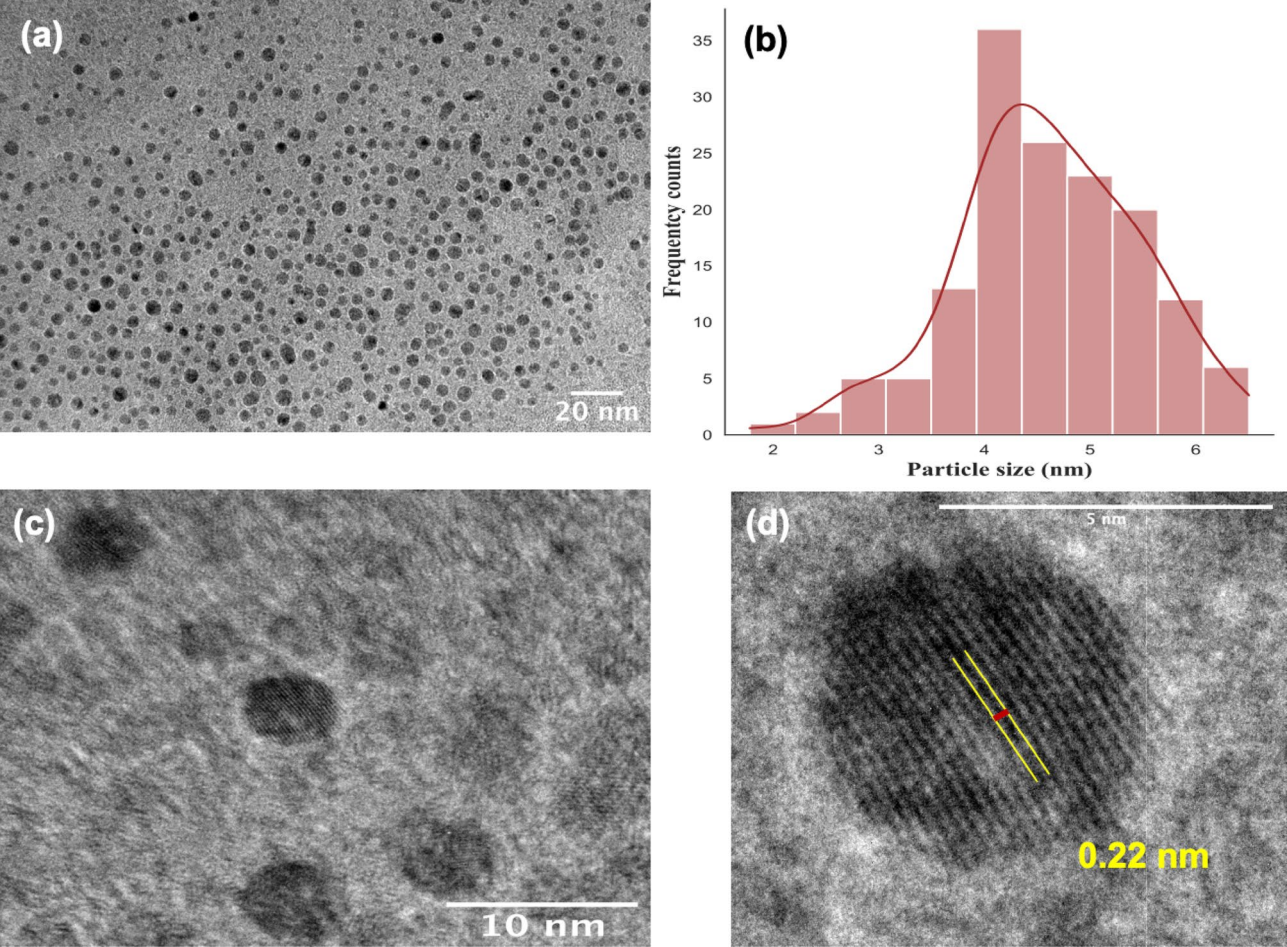
HRTEM images of N-CQDs at different magnification and scale: (**a**) 20 nm, (**c**) 10 nm, (**b**) particle size Gaussian distribution histogram, (**d**) graphitic core lattice. The N-CQDs have commonly a particle size of 4.60 ± 0.87 nm.

HRTEM images of N-CQDs (representative N-0.25 sample) show that the as-synthesized N-CQDs are spherical (Fig. 1a,c) with particle size ranging from 1.78 to 6.50 nm. The Gaussian distribution (Fig. 1b) of a sample of 150 particles shows the mean particle size of 4.60 ± 0.87 nm. In addition, the N-CQDs possess a crystalline structure as indicated by graphite lattice d-spacing of 0.22 nm (Fig. 1d). Similar features were also observed for the other samples g-CQDs, N-1, N-5 and N-10 analysed via TEM (Fig. S3).

**Figure 2:**
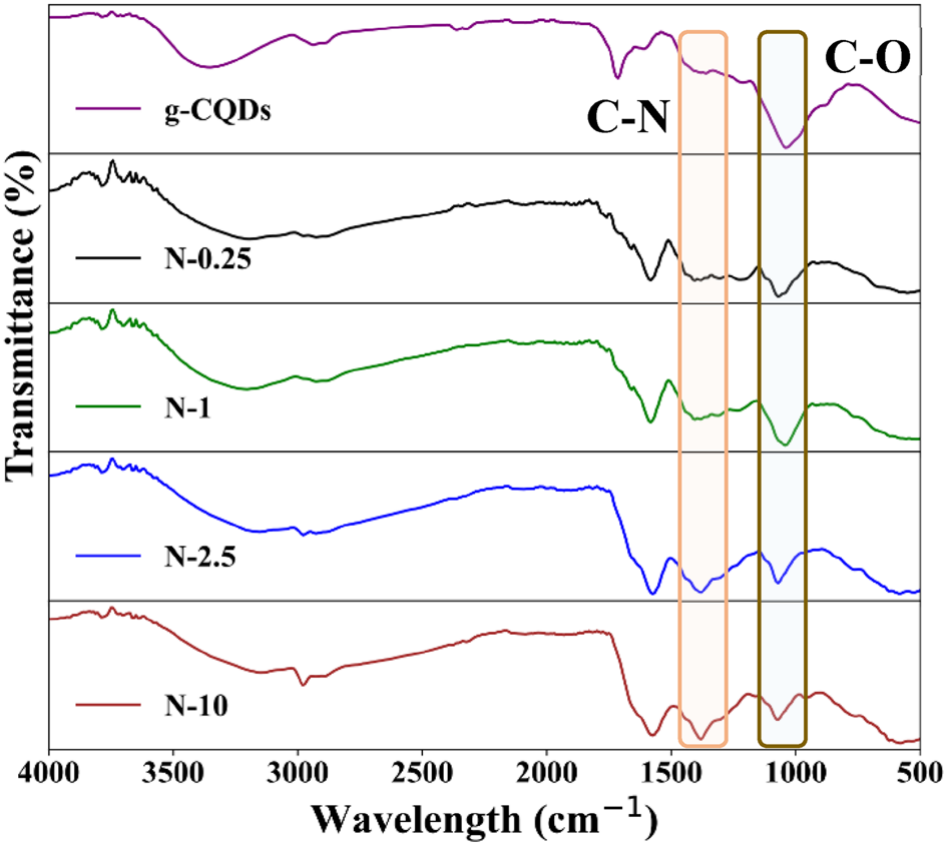
FTIR spectra of N-CQDs with a lower concentration of ammonia (from N-0.25) and higher concentration of ammonia (N-10).

To determine the nature of the functionalisation, the synthesised N-CQDs were investigated using the Fourier transform infrared (FTIR) spectroscopy. The samples were classified into two groups:(i) N-CQDs with a lower concentration of ammonia (from N-0.25 to N-1) and (ii) with a higher concentration of ammonia (from N-2.5 to N-10). The FTIR spectra (Fig. 2) showed that all N-CQDs have hydrophilic groups on their surface such as O–H (hydroxyl) corresponding to the peak at 3389 cm^−1^ and N–H (3263 cm^−1^), thus confirming their good solubility in the water. In addition, vibrations of C–H (2950 cm^−1^), C=O (1581 cm^−1^), C–N (1435 cm^−1^) and C–O (1080 cm^−1^) bonds were also observed in each sample^13–15^. The comparison of the FTIR spectra (Fig. S4) of the samples showed that increasing N-doping (ammonia concentration, from N-0.25 to N-1) displayed a diminishing stretch in vibration for C–O bond at 1080 cm^−1^. While the group of samples with a higher concentration of ammonia (N-2.5 to N-10) showed a sharp vibration of C–N bond at 1435 cm^−1^.

**Figure 3:**
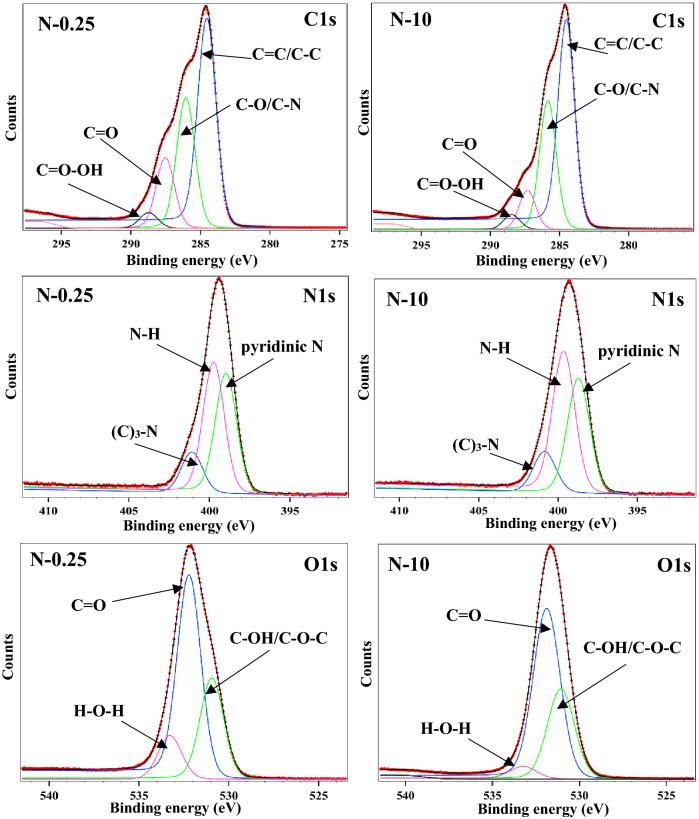
Representative XPS spectra of N-CQDs showing the lowest (N-0.25) and highest (N-10) nitrogen doped samples. The spectra display three typical peaks C1s (285.0 eV), N1s (399.0 eV), and O1s (531.0 eV). The deconvoluted N1s band showed three peaks representing pyridinic N, N–H and amide C–N.

To achieve a deeper understanding of the surface characterisation of the N-CQDs and also to investigate the chemical composition of N-CQDs, X-ray photoelectron spectroscopy (XPS) was employed. The resultant XPS spectra shown in Fig. 3 were deconvoluted using Voigt functions (Lorentzian and Gaussian widths) with a distinct inelastic background for each component^16^. A minimum number of components is used to obtain a convenient fit. The binding energy scale was calibrated to the C 1s standard value of 284.6 eV. The atomic composition has been determined by using the integral areas provided by the deconvolution procedure normalized at the atomic sensitivity factor (Table S1). The XPS spectrum of the N-CQDs displays three typical peaks C1s (285.0 eV), N1s (399.0 eV), and O1s (531.0 eV). The fitted C1s spectrum was deconvoluted into four components, corresponding to carbon in form of C=C/C–C bonds (~ 284.4 eV), C–O/C–N (~ 285.8 eV), C=O (~ 287.3) and O=C–OH (~ 288.4 eV)^17^. Whilst, the N1s band showed three peaks after deconvolution which are 398.8 eV, 399.6 eV and 400.8 eV, representing pyridinic N, N–H and amide C–N, respectively^18^.

The content of each nitrogen doping species (pyridinic, pyrrolic and graphitic) are identified and quantified from the XPS spectra of NCQDs with the purpose of understanding their influence over the optical and chemical properties (Table S2). As commonly reported, the fluorescent property of CQDs can be enhanced by using nitrogen-doping. However, only the nitrogen bonded to carbon can improve the emission^19^. Also, a larger ratio of N/C was observed for N-CQDs samples synthesised with a higher concentration of ammonia (Table S1). The O1s region contains three peaks at 530.9 eV, 532.2 eV and 533.3 eV for C–OH/C–O–C, C=O, H–O–H, respectively^20^. In addition, the oxygen content is also a key parameter in the N-CQDs emission as it can maintain the balance between sp^2^ and sp^3^-carbon atoms^21^. Therefore, Raman spectroscopy was employed to investigate the disorder in the carbon bonding arrangement of N-CQDs.

The Raman spectra (Fig. S5) of the N-CQDs exhibited typical graphitic features consisting of the D mode (at 1368 cm^−1^) related to symmetry transformation by the defects, and the G band (at 1586 cm^−1^), which is assigned to the graphitic core sp^2^ (graphite-like) bonds. This is not surprising as the HRTEM images of N-CQDs showed a typical lattice spacing of graphite (see Fig. 1d). When comparing the Raman spectra between N-CQDs, at first glance, those spectra look similar, a common ratio I_D_/I_G_ of 0.95 revealed a balance between the sp^2^ and sp^3^ bonds in the N-CQDs structure. This is different from g-CQDs where an I_D_/I_G_ ratio of 0.83 was observed and assigned to the carbon core (sp^2^ bonds)^10^. This is probably attributed to the changes introduced by the nitrogen doping resulting in the transformation of C–C (sp^2^ bonds) into the sp^3^ bonding between N, O and C.

### Optical properties of N-CQDs

**Figure 4:**
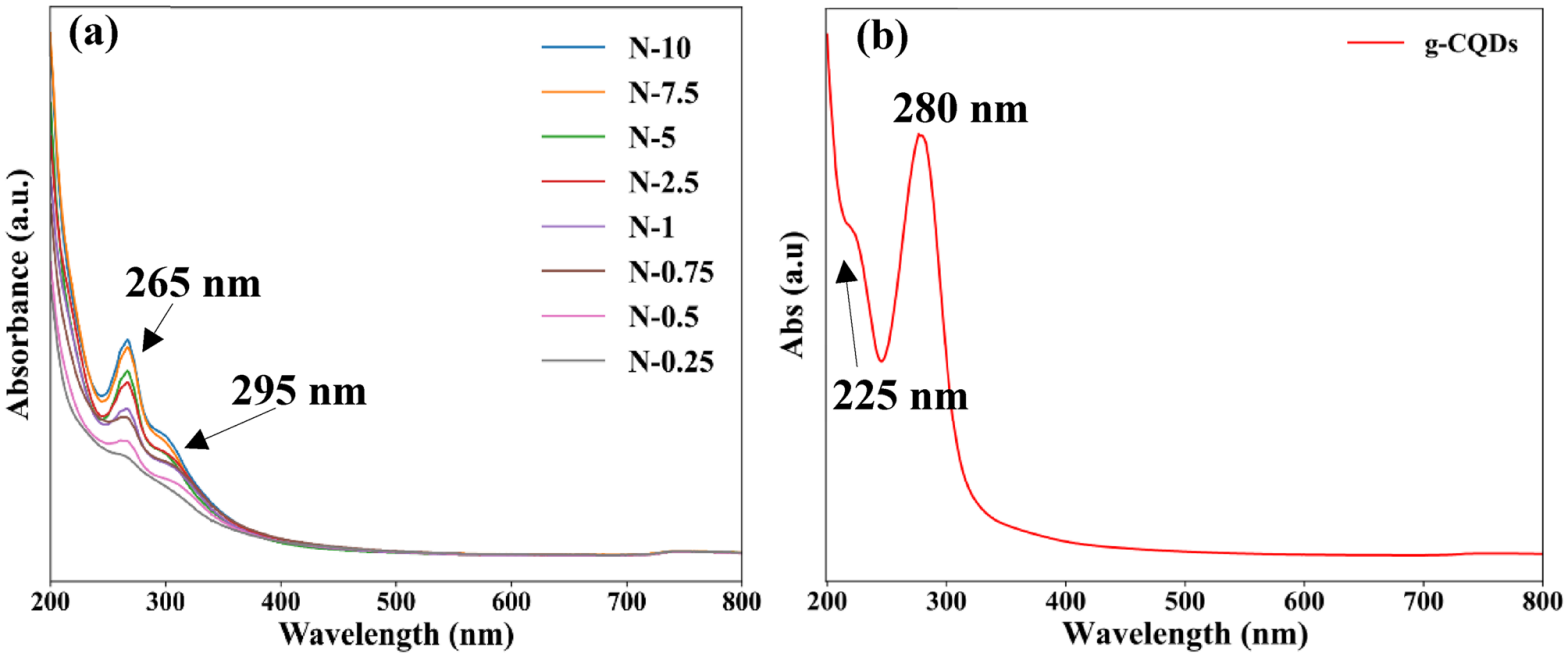
(**a**) UV–Vis absorption spectra of (**a**) N-CQDs and (**b**) g-CQDs without nitrogen doping. The presence of C–N/C=N bonds is observed at 295 nm.

The absorption spectra of the as-prepared N-CQDs measured using UV–Vis spectrophotometry are shown in Fig. 4. The N-CQDs samples have a strong peak around 265 nm and a shoulder around 295 nm (Fig. 4a). The 265 nm absorption peak is characteristics of π–π* transitions of the graphitic core (C=C or C–C) of sp^2^ domains present in the sp^3^ environment, and the 295 nm is attributed to n–π* (C=O) transitions and C–N/C=N bonds^22,23^. For comparison, the absorption spectrum of CQDs without nitrogen doping was also measured**.** It is noted that the absorption peaks related to N-CQDs are red-shifted compared to g-CQDs (synthesised from the same source glucose but without nitrogen doping), Fig. 4b. These transitions, are observed at 225 nm = π–π* (graphitic core), and 280 nm = n–π* transitions (C=O)^10^. Therefore, the absorption peak observed at 295 nm in the case of the N-CQDs is due to the formation of the C–N/C=N bonds related to the doping effect caused by the presence of graphitic nitrogen^24,25^.

**Figure 5:**
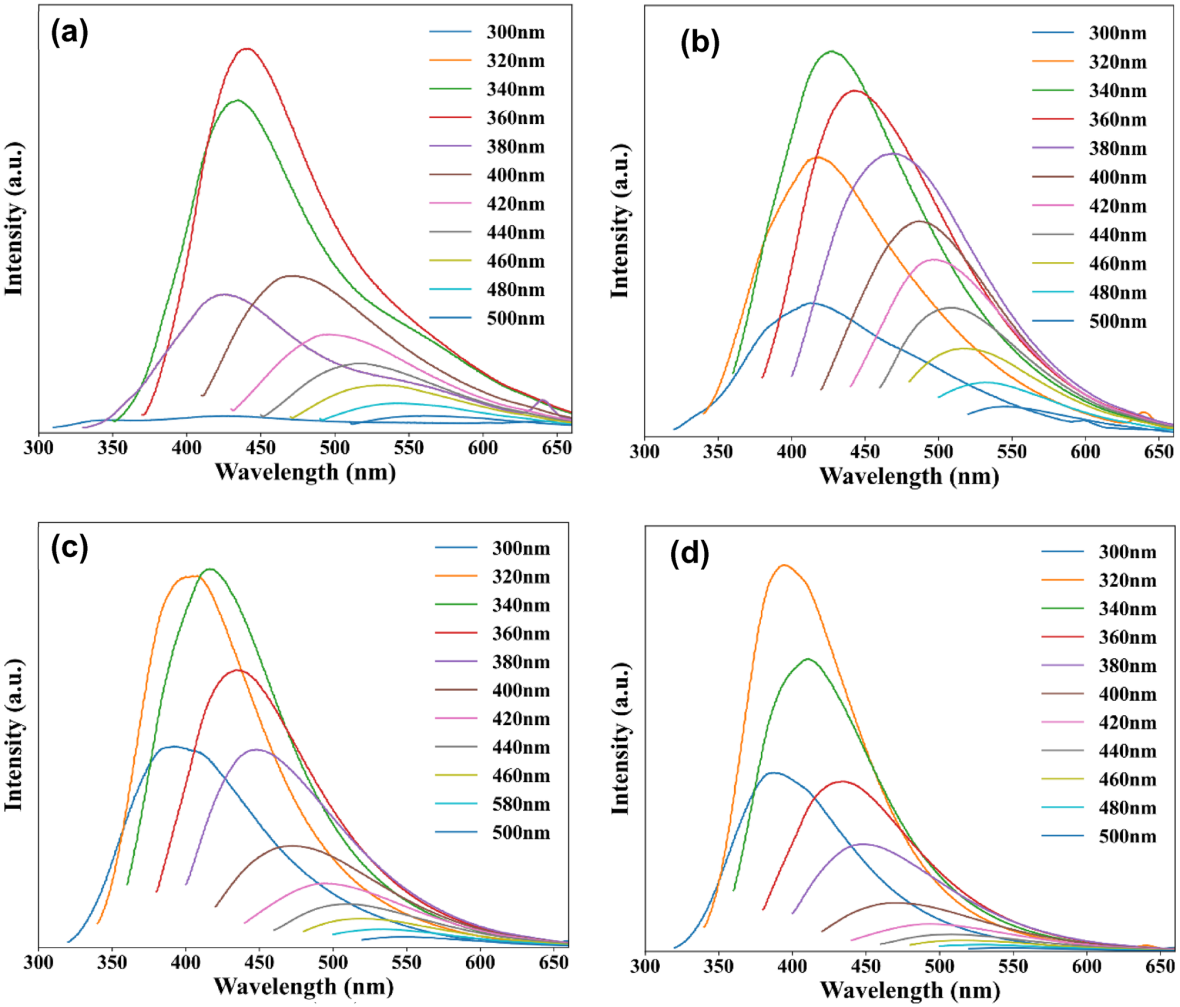
Photoluminescence spectra of CQD with and without nitrogen doping measured using excitation wavelengths in the range of 300 to 500 nm, **(a)** g-CQDs (without nitrogen doping); **(b)** N-0.25; **(c)** N-2.5, **(d)** N-10.

The photoluminescence (PL) spectra of as-prepared N-CQDs were measured using a range of different excitation wavelengths as shown in Fig. 5. The PL emission of each sample clearly showed the excitation-dependent PL which is beneficial for a variety of applications such as biosensors, bio-images, or LED devices^26,27^. The PL emission peaks shifted when different excitation wavelengths were applied, and each sample exhibited an optimal excitation wavelength. Overall, the PL study revealed interesting optical properties of the N-CQDs. Firstly, the PL results are consistent with previous reports where the excitation dependent emission phenomenon of CQDs was observed^28^. Secondly, the maximum excitation wavelengths varied from 360 to 320 nm with the concentration of ammonia.

However, the mechanisms behind the excitation dependent properties of CQDs is not clear. One of the most comprehensive and broadly accepted mechanism in interpreting the excitation-dependent PL of the CQDs is the quantum confinement effect also known as the size effect^14,21,28,29^. In general, the CQDs possess broad particle size distributions which leads to a range of different energy gaps and is the reason for the variation of emission wavelengths^30,31^. But herein, HRTEM image data analyses confirmed that the increased amount of nitrogen doping did not contribute to an increase in the particle size for the as prepared samples. Therefore, the observed red-shift character can be ascribed to the radiative recombination of e−h pairs hosted in the sp^2^ clusters^32^. Aside from the quantum confinement effect, surface states theory is rather broadly adopted to interpret the excitation-dependent PL behaviour of CQDs^33–35^. UV–Vis absorbance showed that the peak of the N-CQDs at 265 nm is related to the π–π* transition, which suggests the existence of a large number of π-electrons. The surface electronic states can conjugate with these π-electrons as a results of the surface oxidation which result in the modification of the electronic structure of the N-CQDs^34,36^.

To interpret the mechanism of this effect, the PL lifetime and PLQY of N-CQDs were measured. The obtained results (Table 1) showed an increase in both PL lifetime and PLQY upon nitrogen doping and the highest values of lifetime and PLQY were obtained for $\left[N\right]\ge 7.5 M$. The obtained PLQY value of 9.6%$\pm$ 0.9 for N-10 is an significant improvement compared to g-CQDs which showed PLQY of < 1%^10^. These results are comparable to the literature (shown in Table S3), where CQDs and N-CQDs were synthesised via different methodologies.

**Table 1:** The photoluminescence quantum yield (PLQY), average lifetime, 1/e lifetime, radiative (k_r_) and non-radiative (k_nr_) rates of N-CQDs.

Sample	PLQY (%)	Average lifetime (ns)	${\tau }_{1/e}$ (ns)	Radiative k_r_ (s^−1^)	Non-radiative k_nr_ (s^−1^)
g-CQDs	0.7 $\pm$ 0.07	4.7 $\pm$ 0.5	2.59	2.7 × 10^6^	3.8 × 10^8^
N-0.25	6.4 $\pm$ 0.6	4.8 $\pm$ 0.5	2.59	2.5 × 10^7^	3.6 × 10^8^
N-0.5	6.6 $\pm$ 0.7	5.4 $\pm$ 0.5	2.98	2.2 × 10^7^	3.1 × 10^8^
N-0.75	6.6 $\pm$ 0.7	5.7 $\pm$ 0.6	3.03	2.2 × 10^7^	3.1 × 10^8^
N-1	6.4 $\pm$ 0.6	6.3 $\pm$ 0.6	3.27	2.0 × 10^7^	2.9 × 10^8^
N-2.5	6.7 $\pm$ 0.7	6.6 $\pm$ 0.7	3.52	1.9 × 10^7^	2.6 × 10^8^
N-5	7.4 $\pm$ 0.7	6.7 $\pm$ 0.7	3.81	1.9 × 10^7^	2.4 × 10^8^
N-7.5	9.3 $\pm$ 0.9	6.7 $\pm$ 0.7	3.81	2.4 × 10^7^	2.4 × 10^8^
N-10	9.6 $\pm$ 0.9	6.5 $\pm$ 0.7	4.69	2.0 × 10^7^	1.9 × 10^8^

The radiative rate (k_r_) and non-radiative rate (k_nr_) were calculated by using the Eq. (2) and (3) ^37^.

2 $${k}_{r}=\frac{\Phi }{{\tau }_{1/e}}$$

3 $$\Phi =\frac{{k}_{r}}{{k}_{r}+{k}_{nr}}$$

where $\Phi$ is PLQY of N-CQD and ${\tau }_{1/e}$ corresponds to the lifetime when fluorescence drops 1/e of its initial value.

**Figure 6:**
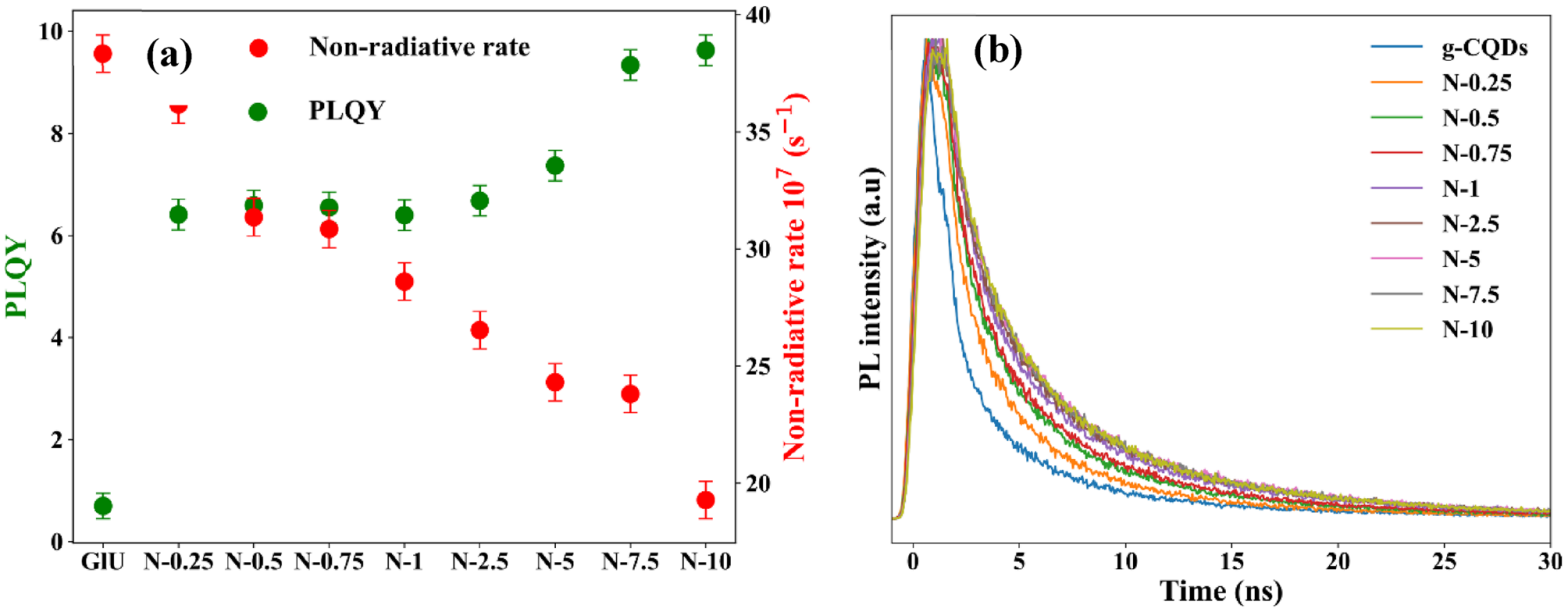
**(a)** PLQY and non-radiative rate (GlU = g-CQDs), (**b)** PL lifetime of g-CQDs and N-CQDs. The analysis revealed an increase in both PL lifetime and PLQY upon nitrogen doping and the highest values of lifetime and PLQY were obtained for [N] ≥ 7.5 M.

Table 1 and Fig. 6 shows that when a higher concentration of ammonia was used, the non-radiative rates significantly reduced. This is due to surface coating activities of the nitrogen functional group which led to enhanced PLQY^38^. In addition, the lower non-radiative constant suggested that N-CQDs possess an efficient recombination process which led to an observation of nanosecond scale PL lifetime. These recombination processes suggested strong coupling of excited core states with the surface state. Thus confirming that the π-electron systems affect the surface electronic state leading to the modification of the overall electronic structure of N-CQDs^36,39^.

**Figure 7:**
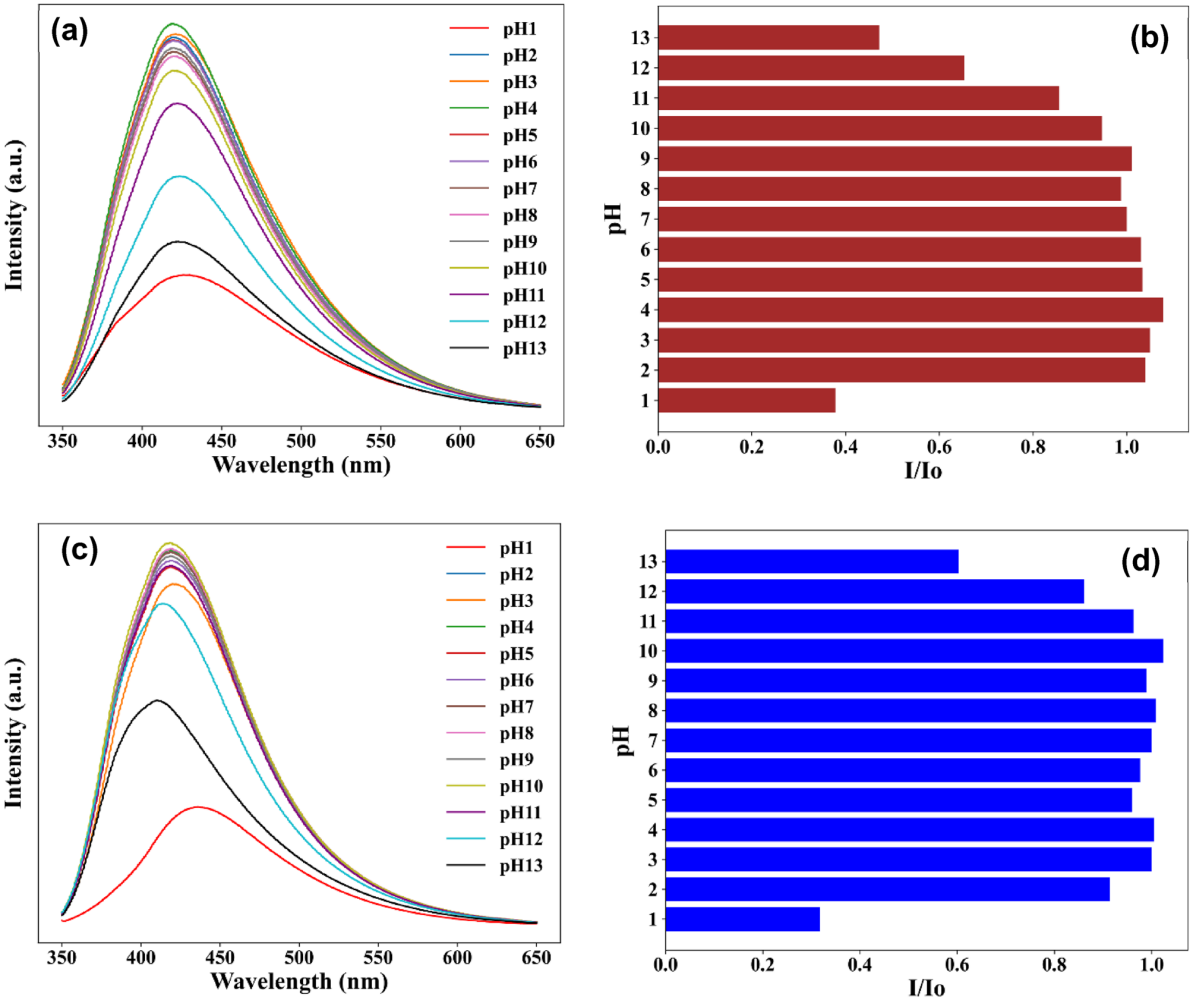
pH effect on the emission intensity of N-CQDs. Representative samples selected to show the highest and lowest [N] doping levels: (**a,b**) N-0.25, (**c,d**) N-10.

The stability of CQDs for a broad pH range is essential for sensing applications. Therefore, PL of the N-CQDs in pH solution was measured to establish the relationship between the pH level and the emission intensity. N-CQDs showed fluorescence stability in a broad pH range from 2 to 11. For example, the fluorescence intensity of sample N-0.25 (Fig. 7a,b) was dramatically reduced by ~ 60% at pH  1, ~ 35% at pH  12 and ~ 40% at pH  13; and there were slight decreases at pH  11 (~ 12%).

The diminishing fluorescent intensity behaviour of N-CQDs in strongly acidic and alkali media, noticeable also for samples with high content of nitrogen (for example, sample N-10, Fig. 7c,d). This can be related to protonation/deprotonation of the surface functional groups which causes surface charge disruption^13^. Whilst H-bonding is eliminated by deprotonation in basic conditions which can cause irregular energy levels resulting in the reduction of the N-CQDs fluorescence^40^. In addition, H^+^ can introduce surface defects on CQDs by breaking the passivated OH^-^ shell resulting in PL decreasing and a redshifted spectrum^41^. Indeed, as shown in Fig. 7c, a 20 nm red shift was also noted for the N-CQD in the strong acidic condition (pH  1). We have previously assigned this to the prominent emissions deriving from the graphitic core^10^.

### Chromium (VI) ion-sensing

**Figure 8:**
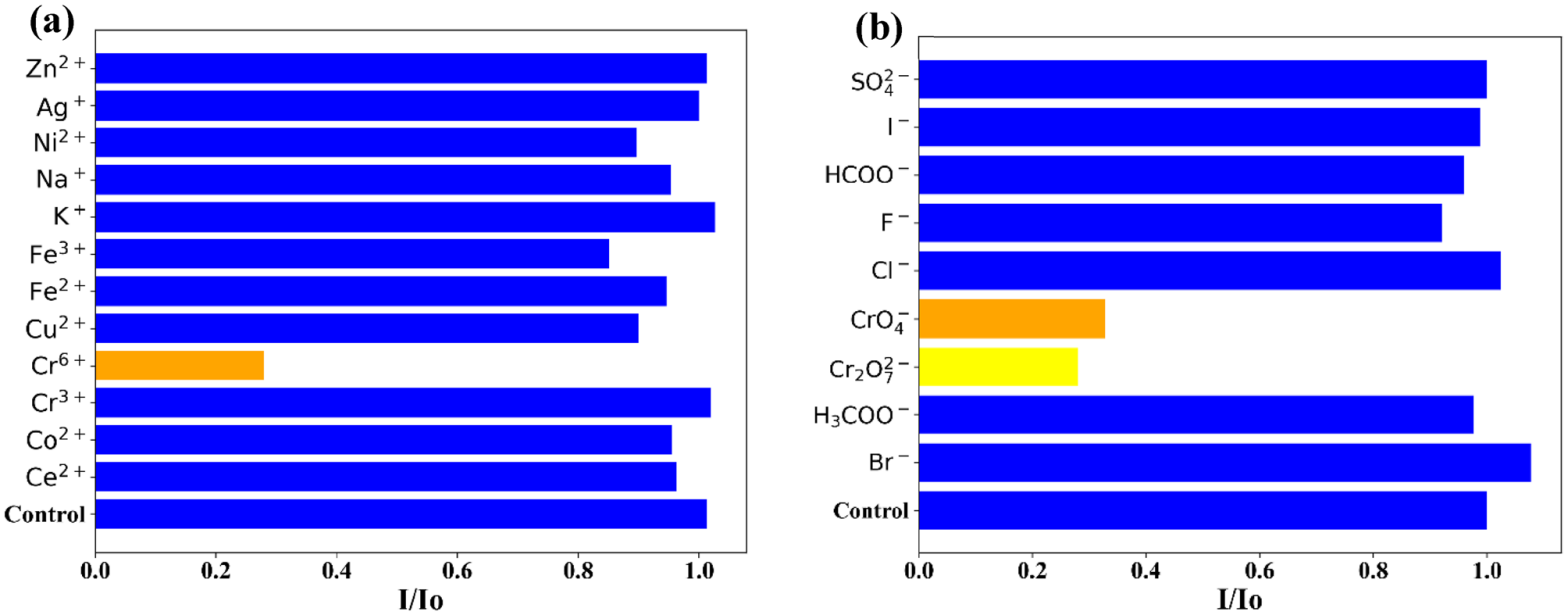
Selectivity of the N-CQDs based chemo-sensor.

The N-CQDs were investigated for ion-sensing applications for a series of cations and anions (Fig. 8 and Fig. S6) including chromium (VI) ion (CrO_4_^2−^/Cr_2_O_7_^2−^) which is a major anthropogenic pollutant in industrial wastewater and soils^11^. The obtained results indicated that N-CQDs are highly sensitive and showed high selectivity towards hexavalent chromium in comparison to a series of other cations and anions.

**Figure 9:**
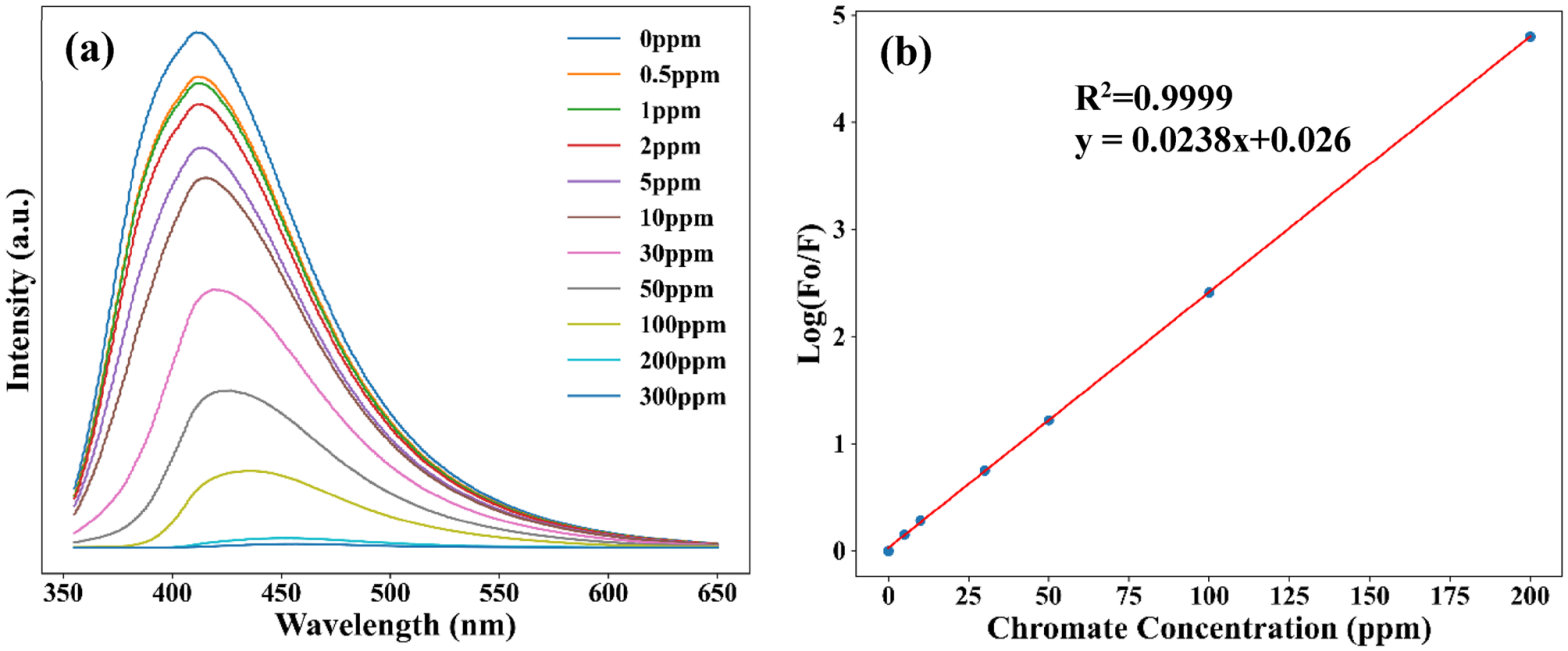
The effect of concentration in the PL intensity of N-10: **(a)** Stem–Volmer graphs as a function of (log(F_0_/F) versus Cr (VI) concentration **(b)**.

**Figure 10:**
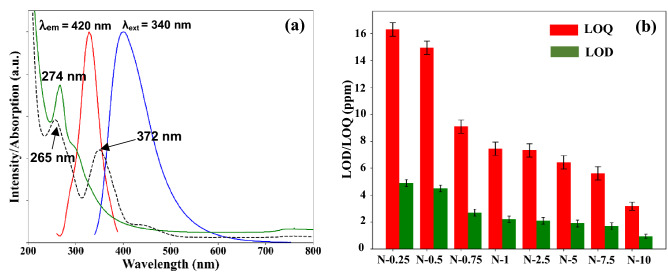
**(a)** Spectral overlap of the normalized UV–Vis absorption bands for the Cr (VI) ions (dash black) and the synthesised N-CQDs (green line), and the excitation spectrum (λ_em_ = 420 nm) (red line) and emission spectrum (λ_ext_ = 340 nm) (blue line) of the N-CQDs**. (b)** LOD and LOQ of N-CQDs.

To identify the sensitivity, limits of detection (LOD) and limits of quantification (LOQ) of N-CQDs were determined by measuring the fluorescent emission quenching as a function of Cr (VI) concentration (Fig. 9a). The reduction in emission intensity with the increase in concentrations of chromium was observed (Fig. 9b) i.e. there is a correlation between them. This correlation was fitted with a linear equation $y=mx+c$, where slope m gives the value of quenching constant K_sv_ and c is the intercept. The LOD and LOQ were determined using the following equations: LOD = 3σ/K_sv_, LOQ = 10σ/K_s_, respectively, where σ is the standard error of the intercept. The plotted Stern–Volmer graph for N-10 in Fig. 9b was fitted with y = 0.0238x + 0.026 (R^2^ = 0.9999) which gave a quenching constant value of K_sv_ = 0. 0238 and intercept of 0.026. The LOD of 0.955 ppm and LOQ of 3.182 ppm were obtained with a standard error of the intercept of 0.0076. The calculated LOD and LOQ for all the samples are shown in Fig. 10. Our results showed a significant improvement compared to previously reported research^10^ where LOD of  3.62 ppm and LOQ of 11.6 ppm) were reported for g-CQDs. In addition, the obtained results are also comparable to other reported literature (Table S4).

To understand the quenching mechanism, the change in PL lifetime of N-CQDs in various ion solutions were studied. The mechanism for this fluorescence quenching behaviour in the presence of Cr (VI) can be assigned to Inner Filter Effect (IFE) which is a physical phenomenon that occurs in a sensing system when the absorption spectrum of the absorber has an overlap with that of excitation and/or emission of the fluorescence leading to the reduced fluorescent emission intensity^13^. The IFE quenching is not related to the radiative and non-radiative transitions in the CQD, thus the intrinsic fluorescence emission is not changed in the presence of the quencher molecule^42^.

As illustrated in Fig. 10a, the N-CQDs excitation and emission bands (λ_ex_ = 340 nm and λ_em_ = 420 nm) overlapped with the chromate (CrO_4_^2−^) anions absorption bands at 372 nm. Moreover, CrO_4_^2−^ shows a second absorption band at 274 nm that also overlaps with the N-CQDs most intense absorption band at 265 nm. Further, the fluorescence lifetime of N-CQDs did not change with the addition of Cr (VI) (Fig. S7 and Table S5) which provides an evidence that the IFE is the mechanism for the fluorescence quenching phenomenon. There is a downtrend of N-CQDs in the LOD and LOQ results (Fig. 10b). The LOD decreased from 4.9 ppm for sample N-0.25 to 0.95 ppm for sample N-10, while the LOQ improved from 16.3 ppm to 3.18 ppm. These results revealed that nitrogen doping enhanced the fluorescent properties of CQDs resulting in higher PLQY which then leads to the improvement in LOD and LOQ and consequently the sensing performances.

## Conclusions

In conclusion, efficient N-CDQs were synthesised using biomass precursors (glucose) and ammonia via the CHFS process. The synthesized N-CQDs possess excellent optical properties with a PLQY of ~ 10% and showed excellent pH stability (for pH 2 to 11). The synthesized N-CQDs were tested as a chemical sensor for Cr (VI) ion and the LOD value of 0.95 ppm and LOQ value of 3.18 nm were obtained. The fluorescence lifetime studies confirmed Inner Filter Effect (IFE) as the mechanism for the quenching behaviour of the nano-sensing. Hence, this work presented a novel, rapid, single-step and green approach for nanomaterials synthesis in general and carbon quantum dots in particular which then can be used for a range of different applications.

## Supplementary Information

Supplementary Video 1.

Supplementary Information.

## Data Availability

The datasets used and/or analysed during the current study are available from the corresponding author on reasonable request. For the purpose of open access, the authors have applied a Creative Commons attribution (CC BY) licence to any author accepted manuscript version arising.
