# Supplementary material for: Investigating the effect of N-doping on carbon quantum dots structure, optical properties and metal ion screening
Source: Sci Rep. 2022 Aug 15;12:13806. doi: 10.1038/s41598-022-16893-x (PMC9378613; doi:10.1038/s41598-022-16893-x)
Supplement: Supplementary file 2 — Supplementary Information. [file 41598_2022_16893_MOESM2_ESM.pdf]

**SUPPLEMENTARY INFORMATION**

# Investigating the effect of N-doping on carbon quantum dots structure, optical properties and metal ion screening

Kiem Giap Nguyen,[a] Ioan-Alexandru Baragau,[a] Radka Gromicova,[b] Adela Nicolaev,[c] Stuart A.J. Thomson,[d] Alistair Rennie,[d] Nicholas P. Power,[b] Muhammad T. Sajjad[a,*] and Suela Kellici[a,*]

[a]London Centre for Energy Engineering, School of Engineering, London South Bank University, 103 Borough Road, London, SE1 0AA, United Kingdom

[b]School of Life Health & Chemical Sciences, Open University, Walton Hall, Milton Keynes, United Kingdom

[c] National Institute of Materials Physics, Atomistilor 405A, 077125 Magurele, Ilfov, Romania

[d]Edinburgh Instruments Ltd., 2 Bain Square, Livingston, EH54 7DQ, United Kingdom

[*]Corresponding authors: *kellicis@lsbu.ac.uk, sajjadt@lsbu.ac.uk; Website: www.nano2d.co.uk*

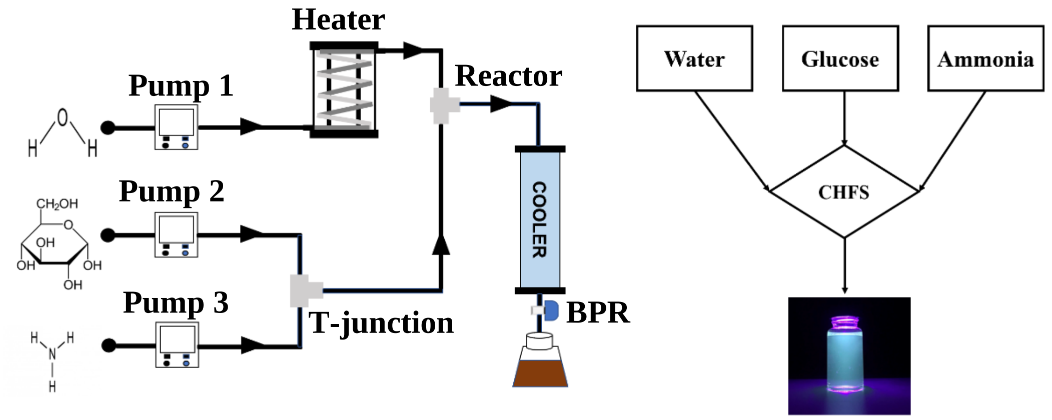


**Figure S1:** *Schematic of Continuous Hydrothermal Flow Synthesis (CHFS) process used to synthesize N-doped carbon quantum dots (NCQDs).*

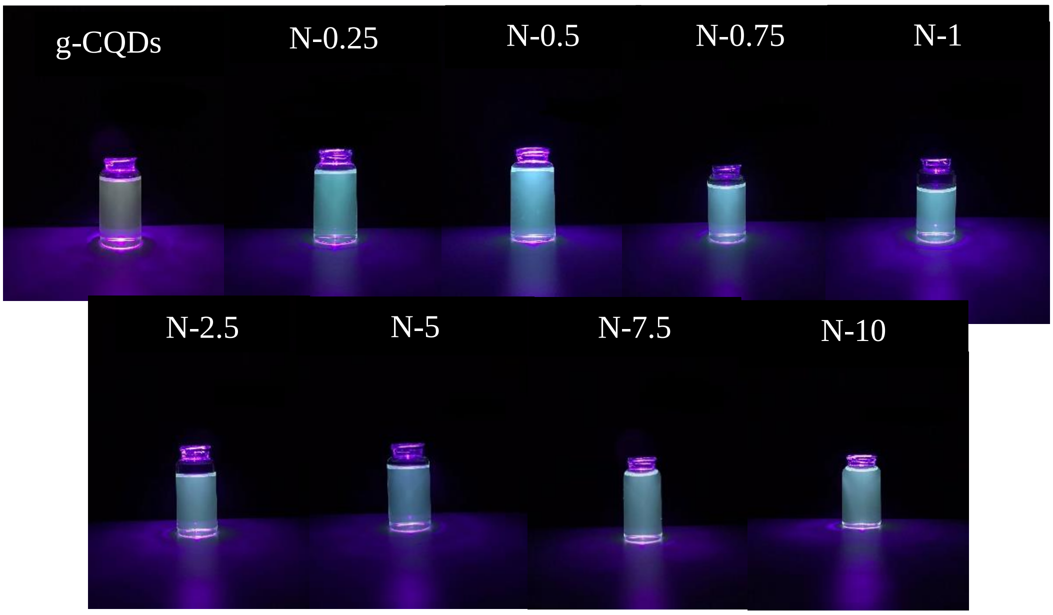


**Figure S2:** *Photos of the synthesised g-CQDs and N-CQDs in water under UV light.*

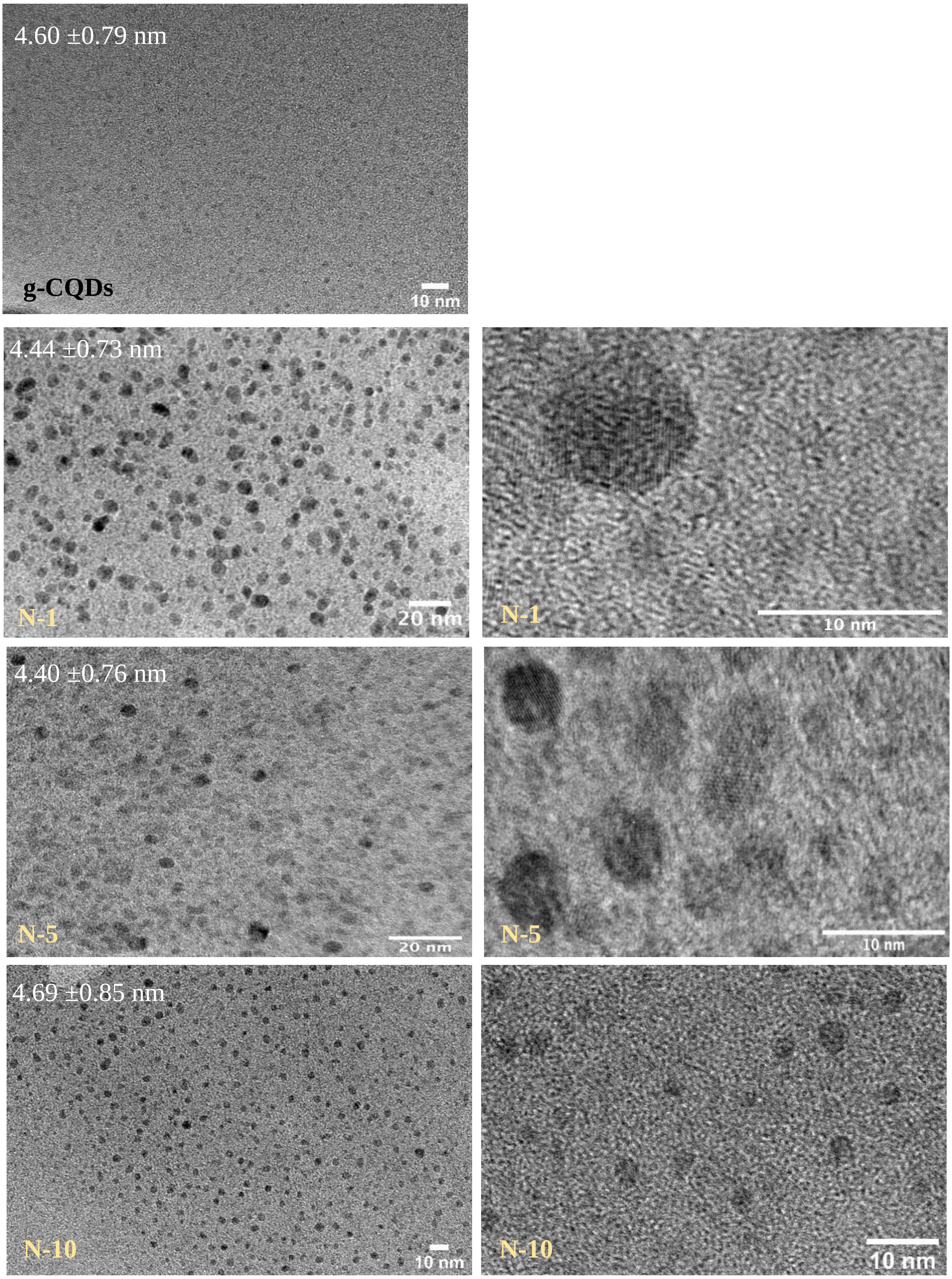


**Figure S3:** *TEM image of g-CQDs, N-1, N-5, and N-10.*

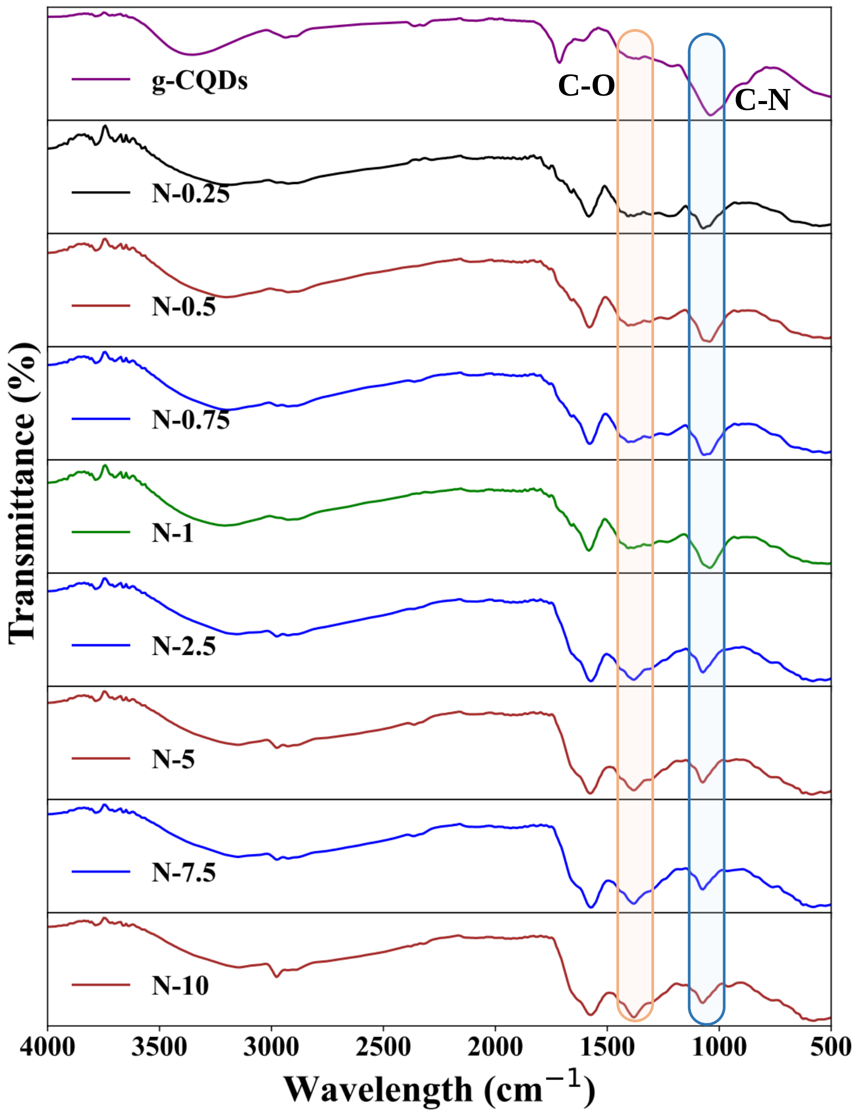


**Figure S4.** FT-IR spectra of the synthesised N-CQDs and g-CQDs

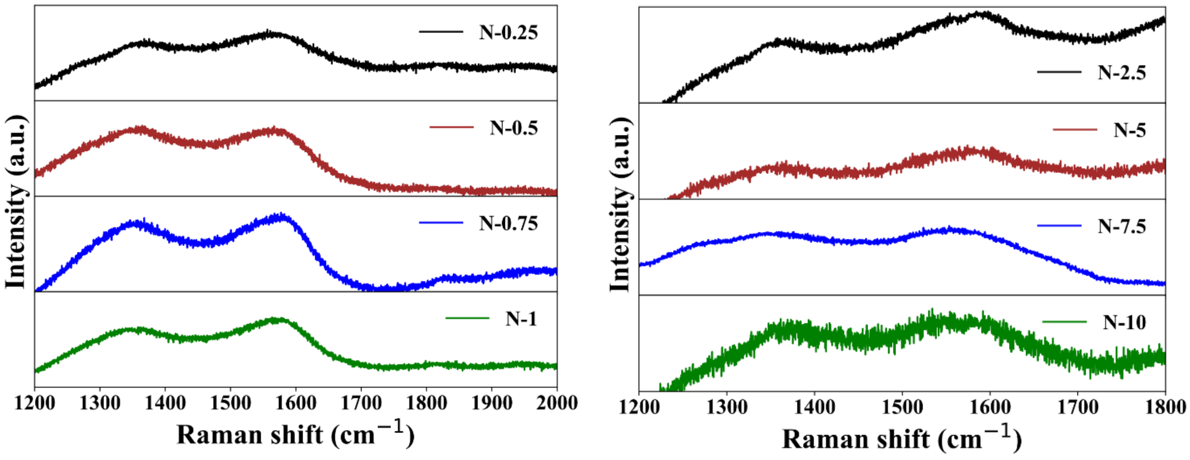


**Figure S5:** *Raman spectra of N-CQDs.*

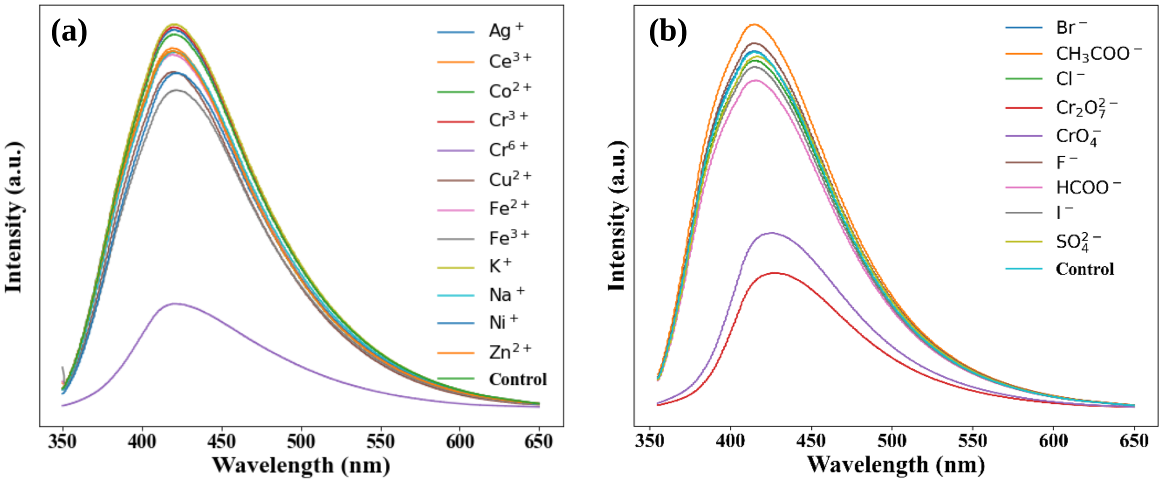


**Figure S6.** *Selectivity of the N-CQDs based chemo-sensor.*

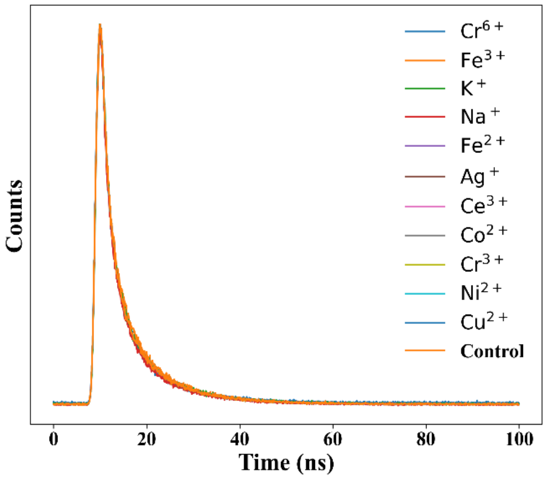


**Figure S7:** *PL lifetime of N-10 in various ion solutions.*

**Table S1.** *XPS data analyses of the elemental composition of N-CQDs*

| **Sample** | **Elemental composition (%)** | | | **N:C ratio** | **C:O ratio** |
|---|---|---|---|---|---|
| | Carbon | Oxygen | Nitrogen | | |
| **N-0.25** | 71.06 | 20.11 | 8.82 | 0.12 | 3.53 |
| **N-0.5** | 71.31 | 20.02 | 8.66 | 0.12 | 3.56 |
| **N-0.75** | 76.11 | 16.3 | 7.57 | 0.10 | 4.67 |
| **N-1** | 68.66 | 24.13 | 7.19 | 0.10 | 2.85 |
| **N-2.5** | 61.8 | 29.6 | 9.22 | 0.15 | 2.09 |
| **N-5** | 67.41 | 19.89 | 12.69 | 0.19 | 3.39 |
| **N-7.5** | 59.39 | 28.96 | 11.74 | 0.20 | 2.05 |
| **N-10** | 57.9 | 31.16 | 10.94 | 0.19 | 1.86 |

**Table S2.** The XPS N1s elemental composition and N1s species content

| Sample | N(1s) content (at%) | Elemental composition N (1s) | | | | | |
|---|---|---|---|---|---|---|---|
| | | Pyridinic | | Pyrrolic | | Graphitic | |
| | | at % | % | at % | % | at % | % |
| **N-0.25** | 8.82 | 3.62 | 41.03 | 4.00 | 44.92 | 1.24 | 14.05 |
| **N-0.5** | 8.66 | 3.46 | 39.92 | 4.00 | 45.53 | 1.26 | 14.55 |
| **N-0.75** | 7.57 | 4.66 | 61.50 | 2.40 | 31.7 | 0.52 | 6.80 |
| **N-1** | 7.19 | 3.04 | 42.25 | 2.80 | 39.15 | 1.34 | 18.6 |
| **N-2.5** | 9.22 | 3.26 | 35.38 | 4.50 | 49.00 | 1.45 | 15.72 |
| **N-5** | 12.69 | 4.64 | 36.53 | 6.20 | 48.70 | 1.87 | 14.77 |
| **N-7.5** | 11.74 | 4.14 | 34.85 | 6.00 | 50.25 | 1.77 | 14.91 |
| **N-10** | 10.94 | 1.51 | 38.90 | 5.20 | 47.24 | 1.51 | 13.86 |

**Table *S3***: PLQY of CQDs and N-CQDs from various methods.

| Carbon resources | Nitrogen resources | Methodology | PLQY (%) | Reference |
|---|---|---|---|---|
| Glucose | L-aspartic acid | Hydrothermal treatment | 7.5 | (1) |
| Glucose | | Hydrothermal treatment | 1.8 | (2) |
| Glucose | | Acidic Oxidation | 1 | (3) |
| Glucose | 4,7,10-Trioxa-1,13-tridecanedi amine | Acidic Oxidation | 13 | (4) |
| Glucose | | Microwave pyrolysis | 3.1 | (5) |

**Table S4**: Comparison of the performance of different materials and techniques.

| Material | Method | Limit of detection (µM) | Reference |
|---|---|---|---|
| Carbon Dots | Fluorescence | 0.25 | (6) |
| Carbon Dots | Fluorescence | 0.26 | (7) |
| Au NPs | Colorimetric | 0.28 | (8) |
| Au decorated/CNTs | Electrochemistry | 0.73 | (9) |
| N-CQDs | Fluorescence | 0.3 | this work |

**Table S5.** Fitting parameter of PL decays of the N-10 in various ion solutions.

| Ion | $\tau_1$ | $a_1$ | $\tau_2$ | $a_2$ | $\tau_{average}$ |
|---|---|---|---|---|---|
| $Cr^{6+}$ | 2.89 | 52.36 | 9.92 | 47.64 | 6.24 |
| $Fe^{3+}$ | 3.08 | 48.65 | 10.62 | 51.35 | 6.90 |
| $K^{+}$ | 2.95 | 51.96 | 10.44 | 48.04 | 6.54 |
| $Na^{+}$ | 2.87 | 52.72 | 10.41 | 47.28 | 6.44 |
| $Fe^{2+}$ | 2.87 | 49.90 | 10.34 | 50.10 | 6.61 |
| $Ag^{+}$ | 2.91 | 54.11 | 10.47 | 45.89 | 6.38 |
| $Ce^{3+}$ | 2.97 | 51.42 | 10.33 | 48.58 | 6.55 |
| $Co^{2+}$ | 2.91 | 53.47 | 10.33 | 46.53 | 6.36 |
| $Cr^{3+}$ | 2.96 | 49.87 | 10.39 | 50.13 | 6.68 |
| $Ni^{2+}$ | 2.99 | 53.93 | 10.57 | 46.07 | 6.48 |
| $Cu^{2+}$ | 3.08 | 50.18 | 10.53 | 49.82 | 6.78 |
| Control | 3.03 | 56.17 | 10.71 | 43.83 | 6.40 |

The average lifetime was computed by using the equation S1:

$$\tau_{avg} = \frac{a_1\tau_1 + a_2\tau_2}{a_1 + a_2} \qquad (eq.S1)$$

where $a_1$, $a_2$, $a_3$ are pre-exponential factors and $\tau_1$, $\tau_2$, are the lifetimes of the fitted components.

**Video S1:** Showing real-time Cr(VI) sensing experiment. As Cr(VI) solution is added dropwise to the blue luminescent NCQDs solution, we observe the quenching of NCQDs luminescence.

## References


1. Zheng M, Ruan S, Liu S, Sun T, Qu D, Zhao H, et al. Self-targeting fluorescent carbon dots for diagnosis of brain cancer cells. ACS Nano. 2015;9(11):11455–61.
2. Cailotto S, Amadio E, Facchin M, Selva M, Pontoglio E, Rizzolio F, et al. Carbon dots from sugars and ascorbic acid: role of the precursors on morphology, properties, toxicity, and drug uptake. ACS Med Chem Lett. 2018;9(8):832–7.
3. Peng H, Travas-Sejdic J. Simple aqueous solution route to luminescent carbogenic dots from carbohydrates. Chem Mater. 2009;21(23):5563–5.
4. Zhu H, Wang X, Li Y, Wang Z, Yang F, Yang X. Microwave synthesis of fluorescent carbon nanoparticles with electrochemiluminescence properties. Chem Commun. 2009;(34):5118–20.

5. Wang L, Li M, Li W, Han Y, Liu Y, Li Z, et al. Rationally Designed Efficient dual-mode colorimetric/fluorescence sensor based on carbon dots for detection of pH and $Cu^{2+}$ ions. ACS Sustain Chem Eng. 2018;6(10):12668–74.
6. Kong W, Wu H, Ye Z, Li R, Xu T, Zhang B. Optical properties of pH-sensitive carbon-dots with different modifications. J Lumin. 2014;148:238–42.
7. Huang Q., Bao Q., Wu C., Hu M., Chen Y., Wang L., Chen W. Carbon dots derived from Poria cocos polysaccharide as an effective" on-off" fluorescence sensor for chromium(VI) detection. J. Pharm. Anal. 2022;12(1):104-112.
8. Gao Y., Jiao Y., Lu W., Liu Y., Han H., Gong X., Xian M., Shuang S., Dong C. Carbon dots with red emission as a fluorescent and colorimetric dual-readout probe for the detection of chromium(VI) and cysteine and its logic gate operation. J. Mater. Chem. B. 2018;6:6099–6107.
9. Guo J.F., Huo D.Q., Yang M., Hou C.J., Li J.J., Fa H.B., Luo H.B., Yang P. Colorimetric detection of Cr(VI) based on the leaching of gold nanoparticles using a paper-based sensor. Talanta. 2016;161:819–825.
